# The Repetition of the Dose

**Published:** 1883-12

**Authors:** Ad. Lippe

**Affiliations:** Philadelphia


### THE REPETITION OF THE DOSE.

AD. LIPPE, M. D., PHILADELPHIA.

If the kind reader expects us to dwell once more on the posological question he will find himself disappointed. With a decided desire to uphold the *liberty* of medical opinión and action, certainly where unsolved questions are at stake, it is best for the extremists to solve them among themselves. We have heard members of the American Institute contend that there exists no medicinal virtue, and, therefore, no healing power, above the 14th or 12th potency; that cures reported to have been made by higher and the highest potencies should not be believed or be allowed to be published.

As illiberal and silly as such assertions are, they have been far surpassed by a member of the International Hahnemannian Association, who not only sets aside the Law of the Similars—the very corner-stone of Homœopathy—but who professes to have *discovered* a new law of cure, viz., that morbific substances (products of a disease) will cure the disease itself *if highly potentized.*

But a useful and practical question commands our attention.

The question, which more than any other, interests and puzzles the true healer, is: "*The Repetition of the Dose.*"

The only positive injunction we find in the writings of Hahnemann is, Never to repeat the dose till the effect of the dose previously administered has been exhausted. When, then, we begin the treatment of an acute or of a chronic disease, we are impliedly commanded to administer *one* dose of medicine. In an acute disease it seems always to be best to give one dose only and carefully observe the results of the action of this one dose. In a great majority of cases this one dose will affect the sick for a very considerable length of time, and in many cases will be all-sufficient to effect by it all the well-chosen remedy can do. What but a detrimental result can follow if too much of a truly homœopathic remedy is given in quick and constant succession? *If* the one dose administered alleviates the case but for a short time, it will only then be proper to repeat it provided always we are perfectly certain that the remedy was truly similar to the case before us, as a repetition will do great harm if it is not the right remedy, and still more harm if it is the right remedy. The burning question remains: How do we know that the remedy administered has exhausted its effects, and is, therefore, to be repeated? It appears to be as much of a conundrum as is the advice of the stockbroker, who gravely tells you that you must always buy at the lowest figure and sell at the highest. Still, we must try and come to a solution of this question—What happens when we administer the truly homœopathic remedy in an acute disease in one proper dose? The very observing healer will often detect a clearly defined demonstration that the remedy has taken effect in a very short time. The more acute and the more grave the attack, the sooner will such evidence become apparent. The groaning of the sick from pain very perceptibly diminishes; the restless, agonizing tossing about will cease, or the overloaded stomach is at once relieved—sometimes so soon (often under the appropriate and health-restoring effects of the homœopathically indicated Nux v., even in the most infinitesimal dose) that, under such circumstances, it is advisable always to provide for the occasion. Or the patient will, very soon after taking the remedy, fall into a good, refreshing sleep or into a perspiration. We are, under such circumstances, assured that we have selected and administered the proper remedy in a proper dose. If, then, the improvement now necessarily following, does cease and the same symptoms remain, it is right and proper to decide that the effect of the dose administered has been exhausted and a new dose is called for. If new symptoms appear (not present when the first dose was administered), these new symptoms are either symptoms belonging to the remedy given or denote the development of the disease. That development may be twofold : it may be the progress of the disease or a critical discharge ; or other symptoms (as a crisis) may show that the recuperative powers of the vis medicatrix naturæ are performing their duty. To decide correctly which of the three here apparent conditions exist requires a full knowledge of our materia medica and of pathology proper. If the new symptoms belong to the remedy, we may safely wait much longer before we may expect that its curative action has been exhausted. If the new symptoms clearly show that the disease is progressing, then not only has that dose exhausted its effects, but is certainly no longer indicated ; it must be dropped. If the new symptoms show that a crisis has set in, then the one dose administered has fairly roused the vital forces to resist the disease, or, better, the disturbed conditions of the organism, and under such circumstances it would be a culpable blunder to interfere in any manner with the now active power of the recuperative forces. If, for instance, in typhus a nose-bleed appears after the administration—say, of Rhus tox.—it would be descending to the role of the symptom coverer if this new symptom should be combated by a multiplicity of internal and local remedies. The true healer never interferes with nature's best efforts to restore health ; the *true healer* knows that secretions of any kind, if they are not abnormal—and thus threatening harm—should never be checked in any possible manner, neither by remedies internally administered nor by external means ; he also knows that such proceedings are always followed by harm. The more the healer knows of the nature and history of the disease before him, the more he appreciates the necessity of treating the symptoms of the sick, and not the disease by name, as such, the better will he be able to decide whether the remedy administered is acting curatively, although a change of symptoms has set in. These new symptoms do denote either an improvement in the condition (a crisis) or a progressive development of it. The close observer will very readily be able to determine what he must do under such circumstances—wait on the effect of the dose administered, or repeat the remedy, or choose another remedy. In periodical diseases, as intermittent fever, or periodically returning pains or spasms and secretions, the reflecting healer will never attempt to break down the paroxysm by repeating even the best selected remedy during such a paroxysm, as the only time to administer the curative remedy is during the time the sick is free from the periodically returning attack.

In chronic diseases we have received additional precautionary advice from Hahnemann himself. After a remedy has for a considerable length of time—sometimes for weeks and months—very much improved the condition of the sick, it not unfrequently happens that the symptoms become worse again. It is not wise at once to repeat the dose, as it very often happens that after a lapse of from five to even seven days the improvement again begins and continues for a very long time, and it is always best to patiently wait at least five days after the improvement ceased before repeating the remedy, and then always in a different potency from the one first given.

In some cases of sickness and under certain circumstances a single dose will benefit the sick for a very long time; in other cases it is necessary to repeat the remedy very frequently for a length of time. The fact is, we must "individualize." If the vital forces are not impaired by previous ill health, palliative treatment, irregular habits, or by age, the single dose will seldom have to be repeated; but where the contrary is the case, then we may expect that it will be necessary to repeat the remedy much oftener. *A priori* we can never determine when, if at all, the repetition of the dose will be necessary.

These reflections have been dotted down with a desire to cause other observing homœopathicians to publish some of their ideas, the results of their experience, and give our journals something to publish in the direction of "true progress" in the healing art left to posterity by its promulgator, Samuel Hahnemann.

